# Metabolic and Endocrine ADRs of Atypical Antipsychotics (AAPs) in Paediatric Patients with Autism Spectrum Disorder (ASD): A Review of Prevalence, Risk Factors, and Implications for Clinical Monitoring

**DOI:** 10.3390/jcm14227942

**Published:** 2025-11-09

**Authors:** Mashal Aljead, Aya Qashta, Zahraa Jalal, Alan M. Jones

**Affiliations:** 1 School of Pharmacy, University of Birmingham, Birmingham B15 2TT, UK; z.jalal@bham.ac.uk (Z.J.); a.m.jones.2@bham.ac.uk (A.M.J.); 2 Psychiatry Services, Brooklands Hospital, Coventry and Warwickshire Partnership NHS Trust, Birmingham B37 7HH, UK; aya.qashta@covwarkpt.nhs.uk

**Keywords:** autism spectrum disorder (ASD), atypical antipsychotics (AAPs), children, adolescents, adverse drug reactions (ADRs), metabolic side effects, endocrine side effects, risk factors, monitoring guidelines

## Abstract

Atypical antipsychotics (AAPs) remain the most effective treatment to control irritability associated with autism spectrum disorder (ASD). Although there is no pharmaceutical treatment to target the core symptoms of ASD, AAPs reduce their severity. However, AAPs have been reported to be associated with severe adverse drug reactions (ADRs) that may lead to long-term conditions such as diabetes mellitus and heart disease. Their prevalence varies depending on the type of AAP prescribed, age, ethnicity, gender, healthcare systems, and the severity of the ASD. Current ADR monitoring guidelines exist, but they are broad in scope and do not fully account for these factors. Therefore, the need to develop ADR monitoring guidelines considering these factors has increased with the expanded use of AAPs in paediatrics with ASD. This gap in knowledge and clinical practice highlights the ongoing need for research to explore these factors and how they can inform the creation of tailored guidelines for monitoring ADRs in this population.

## 1. Introduction

Autism spectrum disorder (ASD) is a neurological condition characterised by challenges in social interaction and repetitive interests [1]. Recently, its prevalence has been reported to be 1% of the population in the UK, 2.5% in Saudi Arabia, and 2.8% in the USA [2,3,4]. The UK and the USA are presented as well-studied populations, whereas Saudi Arabia as an underrepresented population [5]. To date, there is no pharmacological treatment targeting core symptoms (difficulties in social interaction and restricted interests) [6]. However, atypical antipsychotics (AAPs) are more effective at reducing core symptoms than other treatment options, such as antidepressants, stimulants, and mood stabilisers [7]. Additionally, AAPs have been shown to be most effective in managing irritability and comorbidities associated with ASD [8,9]. Furthermore, two AAPs, risperidone and aripiprazole, have been approved by the Food and Drug Administration (FDA) and the European Medicines Agency (EMA) for children aged 5 and older with ASD [10,11]. Risperidone and aripiprazole are therefore the first-line treatments for irritability in children and adolescents with ASD [11]. While other AAPs, including olanzapine, quetiapine, lurasidone, and ziprasidone, are still prescribed for off-label use. These differences in FDA approval status may explain why risperidone and aripiprazole are more commonly prescribed and prioritised in clinical guidelines compared to other AAPs [12]. However, administration of AAPs is associated with neurological, cardiac, metabolic, and endocrine adverse drug reactions (ADRs). Recent studies have focused on metabolic and endocrine ADRs than other ADRs for several reasons. Firstly, they may contribute to the development of long-term conditions, such as diabetes and heart disease [13]. Secondly, they are associated with the risk of osteoporosis and reproductive system issues (e.g., hypogonadism and menstrual irregularities), particularly in children and adolescents [14]. Thirdly, they are easily detected and managed in the early stages, whereas preventing or slowing the progression of their complications can be challenging [15]. These ADRs and their clinical complications are influenced by various risk factors and also impose a burden on healthcare systems [16]. Therefore, investigating the factors affecting ADR prevalence is essential for developing monitoring guidelines for paediatric patients with ASD.

The aim of this review is to explore the factors related to medications, patients, healthcare systems, and disease that influence the prevalence of ADRs, specifically metabolic and endocrine ADRs, and their clinical implications.

## 2. Adverse Drug Reactions (ADRs)

The World Health Organization (WHO) plays a crucial role in ensuring safety for those who are treated with medications and other therapeutic agents by developing clinical guidelines and initiatives. Health organisations worldwide have recently renewed their focus on pharmacovigilance due to the complexity of drug evaluation and the rapid evolution of medications and diagnostic tools [17,18]. WHO defines pharmacovigilance as “The science and activities related to the detection, assessment, understanding, and prevention of adverse drug effects or any other possible drug-related problems” [19,20].

The drug event terminology includes ADRs and medication errors (MEs) [21]. According to WHO, MEs is “any preventable event that may cause or lead to inappropriate medication use or patient harm while the medication is in the control of the healthcare professional, patient, or consumer” [17]. By contrast, WHO defines ADR as “Any response to a drug which is noxious and unintended which occurs at doses normally used in man for prophylaxis, diagnosis, or therapy of disease, or for the modifications of physiological function” [18,22]. In parallel, EMA and the Medicines and Healthcare Products Regulatory Agency (MHRA) in the UK, define ADR as “a response to a medicinal product which is noxious and unintended” [18]. From these definitions, the key difference lies in preventability. To illustrate this explicitly, MEs can be prevented through correct use, while ADRs may occur even when medication is used properly [23].

National and international health organisations encourage health professionals and patients to report any ADRs through national spontaneous reporting systems such as the FDA, the MHRA and the Saudi Food and Drug Authority (SFDA), which in turn analyse the data and report to the Uppsala Monitoring Centre (UMC) [24]. In the UK, ADRs comprised 16% of all hospital admissions, with an average length of stay of approximately one week. The direct cost of ADR admissions for one month was around GBP 468,993 (USD 634,409) in the UK [25]. Similarly, ADRs account for 11.7% of all hospital admissions in the USA, with the average length of stay reaching 11 days. Conversely, in Saudi Arabia, ADRs account for 1.6% of hospital admissions with no prolongation of hospital stay [26].

ADRs are an avoidable strain on the finite resources of healthcare systems. Thus, investigation of these ADRs is essential, particularly in medications potentially associated with severe ADRs or in vulnerable populations such as children or patients with mental health disorders [27]. Based on the classification of Rawlins and Thompson, ADRs can be categorised into six types: A, B, C, D, E, and F [18,28]. Type A (augmented reaction) is dose-dependent and results from the pharmacological activity of the drug, while Type B (bizarre reaction) is idiosyncratic and unrelated to the pharmacological activity of the drug. Type C (chronic reaction) is a time-dependent reaction related to the accumulation of the dose over time, whereas Type D (delayed reaction) appears long after drug use. Type E (end-of-use) occurs upon withdrawal of the drug, and Type F (failure of treatment) refers to unpredictable treatment failure [18,28].

Another scale for ADR classification is the Hartwig Scale (Table 1). It classifies ADRs into seven levels, ranging from mild (levels 1–2) and moderate (levels 3–4) to severe (levels 5–7) [27]. This scale is preferred over other scales for several reasons. Firstly, the Hartwig scale better guides clinical decision-making and patient management by prioritising ADR severity. Secondly, it is more applicable to pharmacovigilance and hospital settings [29]. Thirdly, it is directly relevant to patient safety because it highlights ADRs requiring intervention [30]. This review emphasises levels 3–6. However, level 7 is associated with clozapine administration, which is not used in ASD management [31].

### 2.1. ADRs Associated with AAPs

#### 2.1.1. Metabolic ADRs

Metabolic ADRs encompass metabolic abnormalities that include hyperglycaemia (fasting blood glucose ≥ 110 mg/dL) and hyperlipidaemia (triglycerides ≥ 100 mg/dL or total cholesterol ≥ 170 mg/dL), which contribute to type 2 diabetes and cardiovascular diseases [13]. Children and adolescents receiving AAPs seem to be more prone to metabolic ADRs compared to adults [32]. In particular, children with ASD are more likely to develop metabolic ADR than adults or children with other mental disorders [33,34]. For example, the prevalence of metabolic ADRs reaches up to 55% with AAPs in ASD patients [35].

A recent umbrella review was conducted by Carnovale et al. (2024) [36], evaluating 23 studies (six systematic reviews, 13 meta-analysis, and four networking meta-analysis) to compare the prevalence of metabolic ADRs among AAPs. It found that no AAP showed a statistically significant risk of hyperglycaemia compared to the placebo, as all 95% confidence intervals (CI) crossed zero, indicating a lack of statical significance However, among AAPs, olanzapine (median difference (medianD) = 4.51, 95% CI = −1.70, 10.72) and aripiprazole (medianD = 3.53, 95% CI = −1.66, 8.84) showed comparatively higher effect size on glucose levels, while ziprasidone (medianD = −5.93, 95% CI = −13.23, 1.37) and lurasidone (medianD = 1.67, 95% CI = −3.50, 6.88) showed comparatively smaller effect [36,37]. However, there was no statistically significant difference among these AAPs. Regarding hyperlipidaemia, olanzapine (standardised mean difference (SMD) = 0.40, 95% CI = −0.01, 0.80) showed the highest increase in triglyceride levels, although this was not statistically significant, followed by quetiapine (SMD = 0.37, 95% CI = 0.06, 0.68), which was statistically significant. In contrast, ziprasidone, followed by aripiprazole and risperidone, showed comparatively smaller effect size for hypertriglyceridemia (SMD = 0.09, 95% CI = −0.25, 0.43), (SMD = 0.06, 95% CI = −0.29, 0.42), (SMD = 0.12, 95% CI = −0.07, 0.32), respectively. However, none were statistically significant, as all 95% CIs crossed zero. With respect to weight gain, olanzapine (odds ratio (OR) = 17.34, 95% CI = 3.97, 75.65) posed the highest estimated risk among AAPs, followed by risperidone (OR = 9.01, 95% CI = 2.10, 38.69) and quetiapine (OR = 8.4, 95% CI = 1.58, 44.82), while ziprasidone (OR = 0.45, 95% CI = 0.04, 5.49), followed by aripiprazole (OR = 2.34, 95% CI = 0.47, 11.64), were associated with the lowest estimated risk [36,38]. However, the wide 95% CIs indicate considerable uncertainty that may be due to a small sample size. A study by DelBello et al. (2022) indicated that olanzapine may be associated with an increased risk of weight gain (OR = 44.81, 95% CI = (11.19, 147.70)), while lurasidone appeared to have a lower risk of weight gain (OR = 0.82, 95% CI = 0.22, 2.13) [36,39]. However, the wide 95% CIs indicate considerable uncertainty. The umbrella review demonstrated a wide range and lack of precision in 95% CI findings, which may be due to heterogeneity resulting from the inclusion of studies that use different effect sizes, such as SMD and OR, making direct comparisons and evidence synthesis more challenging. Additionally, the review does not focus specifically on ASD populations, which contributes to inconsistencies in results. Ultimately, the lack of consideration for key demographic factors such as age, gender, and ethnicity limits the generalisability and applicability of the findings.

#### 2.1.2. Endocrine ADRs

Endocrine ADRs are characterised as unintended and harmful effects on the endocrine system that result in abnormalities in hormone secretion, such as hyperprolactinaemia (for <12 years: >25.4 μg/L; for ≥12 years: >18.4 μg/L for males and >24.1 μg/L for females) and thyroid dysfunction (for 2 to ≤17 years; thyroid-stimulating hormone (TSH) is ≥10 mIU/L and thyroxine (T4) is <0.80–1.8 ng/dL) [40,41].

##### Hyperprolactinaemia ADRs

Hyperprolactinaemia is studied more extensively compared to thyroid dysfunction. Although children and adolescents have a higher risk for hyperprolactinaemia, its contributions are primarily examined in adults [42,43]. Hyperprolactinaemia may cause serious complications such as galactorrhoea, menstrual irregularities, infertility, and changes in bone density [14]. The prevalence of hyperprolactinaemia ADR with AAP use was 38.5% in children and adolescents with neurological disorders, including those with ASD [44].

AAPs were linked to significantly increased prolactin levels in children and adolescents compared to placebo (mean difference (MD) = 10.10 μg/L, 95%CI  =  6.97,13.23) [42]. AAPs differ in their risk of inducing hyperprolactinaemia. For example, the prolactin level increases by 28.24 μg/L (*p* < 0.05) and 11.34 μg/L (*p* < 0.01) in risperidone and olanzapine, respectively [42]. By contrast, aripiprazole significantly decreased prolactin to 4.91 μg/L (*p* < 0.01). Other AAPs were not associated with significant elevation, included quetiapine (*p* < 0.06) and lurasidone (*p* = 0.22) [42]. Although ziprasidone is associated with hyperprolactinaemia, the limitations of the studies make it challenging to provide robust conclusions [42]. The main limitations include a small sample for ziprasidone (16 patients), a predominantly male adolescent population (up to 66%), and although the risk is low and unclear, bias could remain, which may limit generalizability.

##### Thyroid Dysfunction ADRs

Quetiapine is the most extensively studied AAP and has been confirmed to cause significant hypothyroidism (*p* < 0.01) with low prevalence [45,46]. Other AAPs lack a significant impact on thyroid function, such as risperidone (*p* > 0.05) and olanzapine (*p* > 0.05) [45]. Due to a lack of controlled evidence supporting the positive correlation between AAP and hypothyroidism, several studies did not demonstrate the need for monitoring serum thyroid hormones in patients with normal thyroid levels hormones [46,47,48]. However, the Canadian Alliance for Monitoring Effectiveness and Safety of Antipsychotics (CAMESA) recommended monitoring thyroid hormones for children and adolescents receiving quetiapine [46,49]. For example, patients with normal TSH levels should undergo repeated testing, while those with abnormal TSH levels require ongoing evaluation monitoring [46]. Additionally, the Maudsley prescribing guidelines recommend annual monitoring of thyroid function in patients taking quetiapine, even though the incidence of thyroid dysfunction is low [50]. Contradictory results have been reported regarding the administration of quetiapine and thyroid dysfunction [46]. For instance, studies by Alvarez-Herrera et al. (2020) [51] and Khoodoruth, Abdo, and Ouanes, (2022) [50] reported elevated TSH levels and a decline in T4 with quetiapine. Meanwhile, the study by Findling et al. (2014) [52] observed no change in T4 and elevation in TSH with quetiapine compared to placebo (*p* = 0.25).

## 3. Risk Factors Influencing the Prevalence of Metabolic and Endocrine ADRs

### 3.1. Medication-Related Factors

#### 3.1.1. Mechanism of Metabolic ADRs

**Figure 1:**
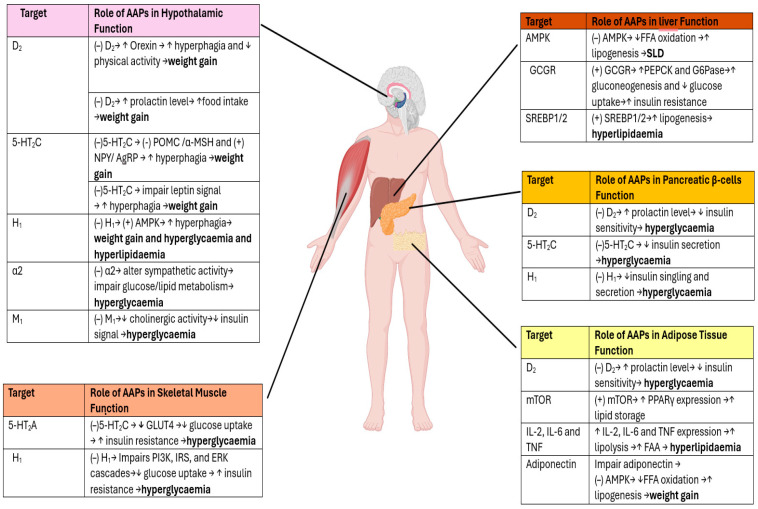
Mechanistic overview of atypical antipsychotics (AAPs)–induced metabolic dysregulation across multiple organ systems. Abbreviation: D_2_: Dopamine; 5-HT_2_A: 5-Hydroxytryptamine (Serotonin) 2A subtype; 5-HT_2_C: 5-Hydroxytryptamine (Serotonin) 2C subtype, H_1_: Histamine, α2: Alpha-2 adrenergic; M_1_: Muscarinic acetylcholine (M_1_ subtype); POMC: Pro-opiomelanocortin; α-MSH: Alpha-melanocyte-stimulating hormone; NPY: Neuropeptide Y; AgRP: Agouti-related peptide; AMPK: AMP-activated protein kinase; GLUT4: Glucose transporter 4, PI3K: Phosphoinositide 3-kinase; IRS: Insulin receptor substrate; ERK: Extracellular signal-regulated kinase; FFA: Free fatty acids; SLD: steatotic liver disease; GCGR: Glucagon receptor; PEPCK: Phosphoenolpyruvate carboxykinase; G6Pase: Glucose-6-phosphatase; SREBP1/2: Sterol regulatory element-binding protein ½; mTOR: Mammalian target of rapamycin; PPARγ: Peroxisome proliferator-activated receptor γ; IL-2: Interleukin-2; IL-6: Interleukin-6; TNF: Tumour necrosis factor; ↑: increase; ↓: decrease. Created in BioRender. Aljead, M. (2025) https://app.biorender.com/illustrations/6807a71b3c36b68f8a93e223.

AAPs induce metabolic ADRs by affecting neurotransmitter signalling (H_1_, D_2_, 5-HT_2_A) or by impairing the function of molecules that regulate appetite, glucose, and lipid metabolism in the central nervous system (CNS) and peripheral organs, including the liver, pancreas, and adipose tissues (Figure 1) [13,15].

##### Weight Gain and Hyperglycaemia

Dopamine receptor D_2_:

The blockage of D_2_ receptors may indirectly induce metabolic ADRs. For instance, D_2_ inhibits orexin, a neuropeptide that regulates appetite and energy homeostasis [53]. When AAPs block the D_2_ receptor, it causes stimulation of orexin expression, resulting in increased hyperphagia and reduced physical activity [15]. Additionally, the prolactin hormone is regulated by D_2_ in the hypothalamus, which affects biological pathways in pancreatic β-cells and adipose tissues [13]. Thus, blocking the D_2_ receptor leads to elevated prolactin levels, decreased insulin sensitivity, and increased food consumption by dysregulating glucose metabolism [54]. Taken together, these mechanisms contribute to weight gain and hyperglycaemia [13,15].

5-HT_2C/2A_ receptors

In the hypothalamus, 5-HT_2_C activates Pro-opiomelanocortin (POMC) neurons to express α-melanocyte stimulating hormone (α-MSH), which is a key peptide in the regulation of appetite as well as glucose and lipid metabolism. AAPs block 5-HT_2_C, causing inhibition of the POMC/α-MSH (anorexigenic) pathway, leading to hyperphagia and decreased fatty acid oxidation and glucose uptake [13,55]. In contrast, AAPs stimulate the neuropeptide Y (NPY)/Agouti-related protein (AgRP) (orexigenic) pathway to induce hyperphagia and weight gain [13]. Additionally, AAPs block 5-HT_2_C in pancreatic β-cells, resulting in a decrease in insulin secretion and hyperglycaemia [13]. Moreover, the blockade of 5-HT_2_C activity disrupts leptin regulation and increases food intake, which leads to weight gain [55,56].

In contrast, AAPs block 5-HT_2_A in skeletal muscles and indirectly impair glucose transporter type 4 (GLUT4), leading to a reduction in glucose uptake by cells [57,58]. This mechanism causes an increase in insulin resistance and hyperglycaemia [13].

H_1_ receptors

One proposed mechanism is that AAPs induce metabolic ADRs through H_1_ antagonism, which indirectly interferes with the AMP-activated protein kinase (AMPK) pathway, which regulates appetite in the hypothalamus. AAPs block H_1_ receptors in the hypothalamus, leading to the activation of AMPK activity, which increases hyperphagia and weight gain [15,59]. In skeletal muscles, AAPs block H_1_ receptors, inducing insulin resistance and impairing insulin signalling by inhibiting phosphoinositide 3-kinase (PI3K), insulin receptor substrate (IRS) proteins, and extracellular signal-regulated kinase (ERK) cascades [15,60]. Furthermore, the activation of the H_1_ receptors in pancreatic β-cells increases insulin secretion [61]. Hence, interfering with insulin signalling or secretion by AAPs contributes to hyperglycaemia and elevation of risk of type 2 diabetes [62].

α2-adrenergic and M_1_ acetylcholine receptors

AAPs modulate α2-adrenergic receptors activity, subsequently altering sympathetic activity. This contributes to increased glucose production and lipolysis, resulting in short-term hyperglycaemia [58,63]. They also act as M_1_ antagonists in the hypothalamus and impair acetylcholine activity in glucose metabolism by inhibiting insulin signalling [64,65].

##### Hyperlipidaemia

All the underlying pharmacological mechanisms of AAPs induce hyperlipidaemia through an indirect effect via the inhibition of neurotransmitter receptors, leading to disruption of glucose and lipid metabolism and insulin resistance [13,15]. However, AAPs also directly affect biological molecules that regulate lipid metabolism [13]. For example, AAPs directly inhibit the AMPK pathway in the liver or do so indirectly through adiponectin signalling in adipose tissue [66]. This leads to the stimulation of lipogenesis by reducing fatty acid oxidation, resulting in the accumulation of lipids in hepatic tissues and weight gain [15,67]. In contrast, AAPs activate the glucagon receptor (GCGR), which stimulates gluconeogenesis by increasing the expression of phosphoenolpyruvate carboxykinase (PEPCK) and G6Pase (glucose-6-phosphat(ase)) while reducing glucose uptake. The activation of GCGR contributes to hyperglycaemia and insulin resistance [15]. Moreover, in adipose tissue, AAPs stimulate the mammalian target of rapamycin (mTOR), which activates lipid metabolism by enhancing the expression of peroxisome proliferator-activated receptor-γ (PPARγ), leading to increased lipid storage [15]. Additionally, AAPs upregulate sterol regulatory element-binding protein 1/2 (SREBP1/2) in the liver, resulting in increased lipogenesis [68]. AAPs also enhance the expression of IL-2, IL-6, and TNF-α in adipose tissue that activates lipolysis and free fatty acids and causes hyperlipidaemia [69,70].

Olanzapine carries the highest risk of metabolic ADRs, followed by risperidone and quetiapine, and then aripiprazole, ziprasidone, and lurasidone [71,72]. Olanzapine carries the highest risk of metabolic ADRs compared to other AAPs, possibly due to its greatest affinity for H_1_ and M_1_ receptors. In contrast, lurasidone presents the lowest risk of metabolic ADRs because it lacks activity on the H_1_ receptor. Consequently, other biological pathways regulated by H_1_, which impact body weight and glucose and lipid metabolism, such as AMPK, PI3K, IRS proteins, and ERK, are not dysregulated by lurasidone [15].

#### 3.1.2. Mechanism of Endocrine ADRs

##### Hyperprolactinaemia

**Figure 2:**
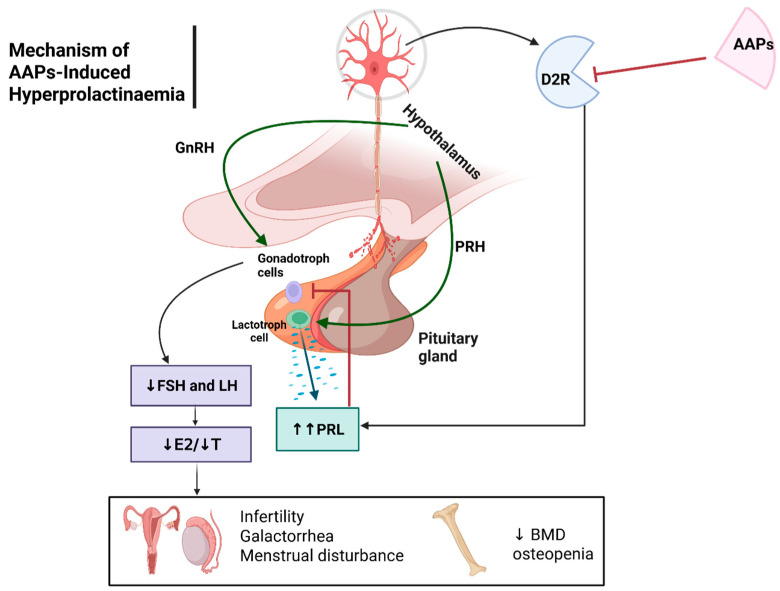
Potential mechanism of AAPs inducing hyperprolactinaemia and their effects on sex organs and bone functions. Abbreviations: D_2_R: Dopamine D_2_ Receptor; AAPs: Atypical Antipsychotics; PRH: Prolactin-Releasing Hormone; GnRH: Gonadotropin-Releasing Hormone; FSH: Follicle-Stimulating Hormone; LH: Luteinizing Hormone; PRL: Prolactin; E2: Estradiol, T: Testosterone, BMD: Bone Mineral Density. ⊥: inhibition; ↶: stimulation; ↑↑: increase; ↓: decrease; Created in BioRender. Aljead, M. (2025) https://app.biorender.com/illustrations/6808f6c4346bb57af605562f.

Hyperprolactinaemia induces menstrual disorders and sexual dysfunction and inhibits bone formation in paediatric patients [41]. This occurs through blockade of D_2_ receptors, leading to elevated prolactin levels. Elevated prolactin levels directly inhibit gonadotropin-releasing hormone (GnRH), which in turn decreases the secretion of follicle-stimulating hormone (FSH) and luteinizing hormone (LH) (Figure 2) [42]. Both FSH and LH play crucial roles in menstrual regulation and fertility. Thus, a decline in these hormones contributes to infertility and sexual dysfunction [73]. Additionally, reduced levels of sex hormones (estradiol and testosterone) interfere with bone mineralization and decrease bone density, particularly in adolescents during peak bone development [74]. The risk of hyperprolactinaemia tends to be increased with risperidone due to its highest affinity for D2 (Ki = 2.29 nM) [75].

##### Thyroid Dysfunction

**Figure 3:**
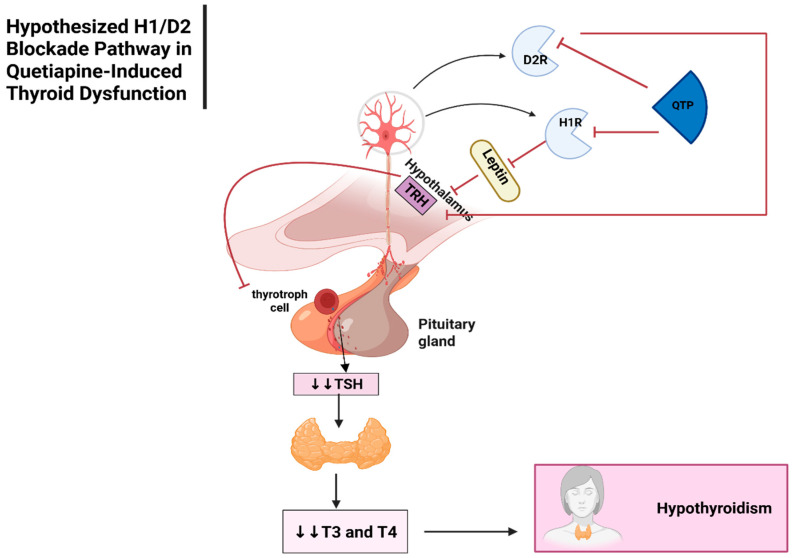
Hypothesised mechanisms related to D_2_ and H_1_ Blockers, including thyroid dysfunction caused by Quetiapine. Abbreviation: QTP: Quetiapine, D_2_R: Dopamine D_2_ Receptor, H1R: Histamine H1 Receptor, TRH: Thyrotropin-Releasing Hormone, TSH: Thyroid-Stimulating Hormone, T3: Triiodothyronine, T4: Thyroxine; ⊥: inhibition; ↓↓: decrease Created in BioRender. Aljead, M. (2025) https://app.biorender.com/illustrations/680a5309a561a9a36a47aa85.

The underlying mechanism of quetiapine induced thyroid dysfunction remains unclear. However, multiple hypothesised mechanisms have been proposed (Figure 3) [50]. The blockade of D_2_ receptors by AAPs may reduce the release of thyrotropin-releasing hormone (TRH) from the hypothalamus, resulting in decreased TSH secretion from the anterior pituitary gland. This leads to decreased triiodothyronine (T3) and T4, causing hypothyroidism [46,50,76]. A major criticism of this hypothesised mechanism, linking D_2_ receptor blockade to reduced TRH and subsequent hypothyroidism, is that quetiapine has a low affinity for D_2_ receptors (Ki = 567 nM) compared to other AAPs [77,78]. If this mechanism were the primary driver of thyroid dysfunction, one would expect higher frequency for this ADR with risperidone, which has the highest D_2_ receptor affinity (Ki = 2 nM) among AAPs [75,77].

Quetiapine has a high affinity for the H_1_ receptor, which could disrupt the hypothalamic-pituitary-thyroid (HPT) axis through alternative neuroendocrine pathways, such as the leptin pathway [79]. However, this proposed mechanism has limitations, olanzapine exhibits higher H1 affinity (2.3 nM) than quetiapine (8.7 nM), which would suggest a higher risk of hypothyroidism. Yet, clinical outcomes differ that may be due to pharmacokinetic factors (e.g., higher c_max_ for quetiapine [1291.4 nM] and differences in tissue distribution compared to olanzapine) and downstream signalling effects rather than receptor affinity alone. Another mechanism associated with quetiapine that induces thyroid dysfunction is the competition between thyroid hormone and quetiapine, and quetiapine being metabolised by uridine diphosphate glucuronosyltransferase (UGT), leading to reduced thyroid hormone levels [76]. This mechanism aligns more closely with olanzapine, for which UGT is one of the primary enzymes involved in metabolism, whereas it plays a secondary role in quetiapine. One further hypothesis considered is that quetiapine stimulates autoimmune thyroiditis, although this has been reported in an inconsistent manner in adults [50,80].

#### 3.1.3. Pharmacokinetic Factors

Risperidone and olanzapine pharmacokinetics (PK) have been the most extensively researched AAP’s in children and adolescents (Table 2) [81].

##### Risperidone

A single oral dose (2 mg) of risperidone is rapidly absorbed and exhibits 70% bioavailability (%*F*), regardless of food intake. Risperidone reaches maximum plasma concentration (c_max_ = 36.5 nM) within one hour and three hours for the active metabolite (9-OH-risperidone) [82,83,84]. It is highly protein bound (90%), with a volume distribution (V_d_) ranging between 1 and 2 L/kg. It exhibits linear pharmacokinetics within its therapeutic dose [56]. Risperidone is primarily metabolised by *CYP2D6*, converting it to its active metabolite (9-OH-risperidone). The half-life (t_1/2_) for risperidone and 9-OH-risperidone is 20 h. The kidneys primarily excrete risperidone, and its clearance significantly declines in patients with renal impairment, causing drug accumulation and an increased risk of ADRs [85].

Generally, in children aged 4 to 15 years receiving risperidone, the pharmacokinetics are similar to those observed in adults [86]. However, the concentration-to-dosage (C/D) ratio of risperidone and 9-OH-risperidone in children is lower than in adolescents and adults [81]. Compared to adults, protein binding is 16% lower in children and adolescents. As a result, the risk of ADRs may be higher in the paediatric population due to an increased free fraction (26%) of risperidone [87].

The pharmacokinetics of risperidone are influenced by several factors, including concomitant medications and *CYP2D6* phenotype. The co-administration of risperidone with *CYP2D6* inhibitors (e.g., fluoxetine, valproate) reduces the metabolism of risperidone and increases the level of exposure by up to 55%, resulting in an increased risk of ADRs [81]. Moreover, the metabolism of risperidone is influenced by the *CYP2D6* phenotype, which may lead to variability in the prevalence of ADRs due to differences in drug metabolism activity across populations and ethnicities [81,88,89]. In the study by Biswas, Vanwong, and Sukasem (2022) [31], weight gain and hyperglycaemia ADRs are reported among poor metabolizers (PMs). Therefore, dose adjustment of risperidone should be considered for patients who are PMs or those who concomitantly take *CYP2D6* inhibitors [88].

##### Aripiprazole

Aripiprazole is well absorbed with or without food, showing an 87% bioavailability [90]. It exhibits linear pharmacokinetics at therapeutic doses, reaching c_max_, which is approximately 240.8 nM, within 3–5 h after 2–15 mg of oral aripiprazole [91,92]. It has a V_d_ of around 5 L/kg with 99% plasma protein binding, predominantly to albumin [56]. It is mainly metabolised by *CYP2D6*, producing the active metabolite (dehydroaripiprazole) and inactive metabolite (aripiprazole metabolites-1451) [81,93]. The aripiprazole t_1/2_ is 75 h and becomes longer by 20% in dehydroaripiprazole. Aripiprazole is excreted mainly via faeces (55%) and urine (30%) [56].

Aripiprazole pharmacokinetics are comparable between children aged 10–17 years old and adults [94,95]. Nonetheless, factors such as gender and enzyme phenotype play a crucial role in the variability of aripiprazole pharmacokinetics [96,97]. Regarding gender differences, at an equivalent therapeutic dose of aripiprazole, the c_max_ was 11% higher in females than in males (*p* < 0.05) [97].

There is no significant correlation between the concentration of aripiprazole in plasma and the co-administration of *CYP2D6* inhibitors. However, this concentration is influenced by the *CYP2D6* genotype [98,99]. For example, the t_1/2_ is doubled in PM, reaching 150 h compared to extensive metabolizers (EMs) [93,96].

##### Olanzapine

Olanzapine is well absorbed after oral administration, and its absorption is not affected by food. It exhibits linear pharmacokinetics, with a bioavailability of ≥65% [32,100,101]. Its c_max_ is 48 nM after oral administration [102]. It binds approximately 93% of albumin and α1−acid glycoprotein and has a V_d_ of 21 L/kg [103]. Regarding its metabolism, *CYP2D6*, *CYP1A2,* and *UGT1* convert olanzapine into three inactive metabolites, including 2-hydroxymethylolanzapine, *N*-desmethylolanzapine, and 10′/4′-*N*-glucuronides, respectively [100,103,104]. The t_1/2_ of olanzapine ranges from 30 to 52 h, with an average clearance rate of 25 L/h. Up to 57% of olanzapine is renally excreted, while the remaining amount is excreted through faeces [100,103].

Factors such as age, gender, and concomitant medications that may affect the pharmacokinetics of olanzapine have shown conflicting results. Studies indicated that the plasma concentrations of olanzapine differ between adolescents and adults [32,81,101,105]. In contrast, several studies reported that a negative association was identified between age and plasma concentration of olanzapine [81,106]. These divergent outcomes may result from overlooking the factors influencing the pharmacokinetic profile. For instance, the t_1/2_ and c_max_ in adults are comparable to those observed in children aged 10–18 years old [107]. By contrast, clearance is reduced by 48% in younger children (those under 12 years old), leading to a 43% increase in olanzapine exposure compared to adults [101,108]. Additionally, females tend to have lower *CYP1A2* activity than males, contributing to a 30% reduction in clearance. Consequently, both children and females may require a lower dose of olanzapine than adult males [32,81]. On the other hand, the co-administration of *CYP1A2* inducers, such as cigarette smoking, increases olanzapine clearance by 40% in patients who smoke tobacco or e-cigarette (vaping) [109,110].

##### Quetiapine

Oral quetiapine is rapidly absorbed and is not affected by a low-fat meal, showing approximately 10% bioavailability and demonstrating linear pharmacokinetics [111,112]. Following 300 mg of quetiapine, the c_max_ was observed to be 1291.4 nM [113]. Its V_d_ varies from 6 to 14 L/kg, and albumin binding reaches 83% [111,114]. It is extensively metabolised by *CYP3A4*, which converts it to the active metabolite. *N*-desalkyl quetiapine [115]. The t_1/2_ of quetiapine is approximately 6 h, with an elevation of 12 h for *N*-desalkyl quetiapine [114]. Quetiapine is excreted through urine and faeces, accounting for 73% and 20%, respectively [111].

The influence of age on quetiapine pharmacokinetics has been reported in several studies [81,116,117]. For example, the plasma concentration of quetiapine is 41% higher in adults compared to children aged 10–17 years old [117]. In contrast, in the study by McConville et al. (2000) [118], the t_1/2_ and c_max_ in adults are similar to those observed in children aged 12–17 years. Specifically, this discrepancy between these findings may be due to variations in study design, sample size, and age range of participants. The strength of this study is due to the systematic, population-based pharmacokinetics data across a wide paediatric age range. Meanwhile, the open-label trial conducted by McConville et al. (2000) [118] had smaller sample sizes that followed DSM-IV criteria and provided limited pharmacokinetic detail, making them less suitable for modelling or dose optimisation.

The pharmacokinetics of quetiapine are influenced by concurrent medications, CYP3A4-dependent factors, and hepatic impairment [119,120,121]. The administration of 400 mg/day of quetiapine with *CYP3A4* inhibitors (e.g., erythromycin) increases its t_1/2_ by 92%. Therefore, a six-fold dose reduction is essential for the co-administration of quetiapine with *CYP3A4* inhibitors [120,121,122]. The quetiapine dose is reduced by 10% to 50% due to decreased clearance in patients with hepatic impairment [121]. There is a lack of evidence to support a gender difference with quetiapine [123].

##### Lurasidone

The absorption of lurasidone increases by 2 to 3 times when taken with food [124]. Its c_max_ is 60.9 nM after a 20 mg dose with bioavailability varying between 9 and 19% due to first-pass metabolism [124,125,126]. Lurasidone binds approximately 99% to plasma proteins, with V_d_ varying between 2.4 and 20 L/kg [127]. It is primarily metabolised by *CYP3A4*, producing two active metabolites, namely *exo*-hydroxy metabolite ID-14283 and *endo*-hydroxy metabolite ID-14326 [115]. The average t_1/2_ of lurasidone is 29 h as the parent drug and becomes 2.5-fold shorter in ID-14283. Approximately 80% of lurasidone is excreted in faeces, while 9% is excreted in urine [128].

Numerous studies show that pharmacokinetics (e.g., plasma concentration) negatively correlates with age [129,130,131]. However, both gender and concurrent medication influence its pharmacokinetics. In the study by Yang et al. (2022b) [132], it was reported that the area under the curve (AUC) of lurasidone is approximately 15% higher in females than in males. In relation to concurrent medications, prescribing lurasidone (40 mg/day) with *CYP3A4* inhibitors (e.g., ketoconazole 400 mg/day) increases AUC by approximately ten times and C_max_ by seven times compared to lurasidone alone [72,133]. Consequently, it is advisable to avoid co-administration of ketoconazole with lurasidone and switch to an alternative antifungal [72].

##### Ziprasidone

Ziprasidone is well absorbed, and its c_max_ reaches 121 nM within 6 h after a 20 mg dose [134,135]. The bioavailability of ziprasidone is approximately 60% and increases to 100% when consumed with food [136]. It is 99% bound to plasma albumin and α1 acid glycoprotein. It has a V_d_ of ~1.5 L/kg. The majority of ziprasidone’s metabolism is mediated by glutathione and aldehyde oxidase, while *CYP3A4* plays a lesser role [72,115]. Ziprasidone’s t_1/2_ is approximately 7 h, with the main route of elimination being faecal (66%), while the remainder is excreted in the urine [137].

The pharmacokinetics of ziprasidone in children are comparable to those in adults [81,138]. Several factors, including gender and polypharmacy, play a critical role in ziprasidone pharmacokinetics. The c_max_ of ziprasidone is approximately 25% higher in females than in males, which is statistically significant (*p*  <  0.05) [123]. Despite *CYP3A4* having a minor effect on ziprasidone metabolism, the co-administration of 400 mg/day of ketoconazole significantly increases the AUC and c_max_ by approximately 34% for a 40 mg/day dose of ziprasidone (*p* < 0.05) [72,139].

### 3.2. Patient-Specific Factors

#### 3.2.1. Age

Children and adolescents, particularly those with ASD who are administered AAPs, have a greater prevalence of metabolic and endocrine ADRs compared to adults [32,34]. There are multiple reasons that may explain why paediatric populations are more likely to develop these ADRs. As noted, their physiological systems for metabolising and eliminating medications are not as fully developed as those in adults [81,140].

In children, gastric *p*H is initially higher and gradually decreases to reach adult levels in the second year of childhood due to an immature digestive system. This can affect the absorption of AAPs [141]. For distribution, body composition changes by decreasing extracellular water and increasing fat storage throughout childhood. This causes a continuous change in V_d_ of drugs and t_1/2_, necessitating adjustments in dosage for AAPs [101,142]. In addition, paediatric populations have lower plasma protein levels compared to adults. For example, AAPs are highly protein-bound, leading to increased free AAP concentrations and a higher risk of ADRs in children [141].

In addition, historically, children have been insufficiently included in clinical trials, which results in limited paediatric studies and creates challenges in determining safe and effective doses for children [141,143]. Furthermore, during puberty, these differences become more pronounced as hormonal fluctuations influence the concentration and clearance of the drug [81,141].

#### 3.2.2. Gender

With respect to gender variation, female adolescents who are receiving risperidone or aripiprazole are more prone to metabolic ADRs, particularly weight gain, than males [144]. Furthermore, a statistical difference in the prevalence of weight gain (i.e., the increase in body mass index (BMI) was 1.3–1.5 kg/m^2^) was observed between males and females receiving AAPs (i.e., aripiprazole, olanzapine) (*p* < 0.001) [97,145]

Generally, the prevalence of endocrine ADRs, particularly hyperprolactinaemia, is 20% higher in females than in males who are receiving AAPs (i.e., risperidone, olanzapine, quetiapine, ziprasidone, and lurasidone), which may be due to the estrogenic effect on prolactin levels [42]. Another study showed that females receiving AAPs (i.e., aripiprazole, olanzapine, and quetiapine) experienced a higher rate of thyroid dysfunction ADRs compared to males, although the difference was not statistically significant (*p*  = 0.134) [146].

There are several hypotheses for the variation in the prevalence of ADRs between males and females [147]. In the past, females were not included in clinical trials due to concerns about the teratogenic risk to those who could become pregnant [148]. Additionally, females typically have lower body mass and a higher proportion of adipose tissue, which influences the absorption and distribution of medications [149]. Furthermore, females exhibit a lower glomerular filtration rate (GFR) and P-glycoprotein (P-gp) renal efflux of medication compared to males, resulting in a prolonged t_1/2_ [150,151]. Moreover, females report ADRs more often than males [152]. Therefore, gender differences should be considered in guidelines to reduce the risk of ADRs.

#### 3.2.3. Ethnicity

Ethnicity also plays a crucial role in determining the prevalence of metabolic and endocrine ADRs among AAPs. The Caucasian group is overrepresented compared to other ethnicities, such as Middle Eastern populations, which limits the generalizability of these study findings [153].

This lack of diversity is particularly significant when considering pharmacogenetic factors that influence drug metabolism. AAPs are primarily metabolised by three key enzymes: *CYP2D6*, *CYP3A4*, and *CYP1A2* [154]. The activity of these enzymes varies across ethnic groups due to differences in the frequency of genetic variants, which can, in turn, affect the efficacy and safety profiles of AAPs [129].

Regarding *CYP2D6*, the metabolites OH-risperidone and dehydroaripiprazole decrease approximately two times in PMs and one and a half times in intermediate metabolizers [155,156]. A cohort study showed that *CYP2D6* PMs of risperidone are most prevalent in Oceania (21%). In contrast, in the Middle East, *CYP2D6* PMs rate is around 12.7%, followed by Europe (9.3%) and America (8.4%). While *CYP2D6* PMs of aripiprazole are most prevalent among Europeans at 6.1%, the rate in Americans is 3.7%, and in Middle Easterns, it is 1.2% [157]. This indicates that the risk of risperidone ADRs is most frequently observed in Oceania, followed by the Middle East, Europe, and America. In contrast, the risk of ADRs associated with aripiprazole appears to be higher in Europe than in the Middle East populations. According to the Dutch Pharmacogenetics Working Group (DPWG) guideline, in *CYP2D6* PMs, the dose of risperidone and aripiprazole should be prescribed at 50–67% of the therapeutic dose to reduce the risk of ADRs. In contrast, in ultrarapid metabolizers, risperidone should be switched to other medications to prevent treatment failure [157]. Interestingly, the rate of *CYP2D6* PMs in the East Asian population is between 0.9% and 1.2% for aripiprazole and risperidone, respectively. This may explain why the therapeutic dose of risperidone in East Asians is reduced by 26% compared to Europeans (around 50%) [157,158,159].

With reference to *CYP3A4* PM, quetiapine plasma concentrations were observed to be more than three times higher. Therefore, they need a dose reduction to less than a third or a switch to an alternative medication with lower *CYP3A4* dependence based on clinical use [119,154]. *N*-Desalkyl quetiapine exhibits antidepressant effects and is present in reduced concentrations in PM. Therefore, switching to an alternative drug is highly recommended for treating depression in PMs [154].

In terms of *CYP1A2*, there is no significant correlation between olanzapine exposure and its phenotype. Numerous clinical studies did not observe any difference in the prevalence of ADRs across different populations [100,160,161]. Therefore, the DPWG recommended that no further action be taken [154].

### 3.3. Healthcare System Factors

The differences in strategies and policies for reporting and managing ADRs are among the key factors that contribute to variations in ADR prevalence between countries [162]. For instance, the MHRA partnered with patient organisations to develop pharmacovigilance guidelines and enhance patient awareness [163]. Similarly, the SFDA collaborated with the WHO to enhance ADR reporting among patients [164]. In contrast, in some countries, the absence of guidance for patients on reporting ADRs has led to a decline in the number of reported cases [165]. Moreover, some countries, such as the UK, standardise ADR reporting for both medications and herbal medicines, while Taiwan uses different forms that may delay responses from healthcare professionals (HCPs) [163]. Furthermore, the use of information technology in ADR reporting has increased to improve the validity and reliability of ADR data and to speed up response times for curating, managing, and analysing ADRs [166]. However, this facility remains limited in low-income countries, resulting in a misrepresentation of ADR prevalence globally [167].

### 3.4. Disease-Related Factors

As described, ASD is a heterogeneous disorder that produces varying severity of symptoms and sensitivity to medications, resulting in increased susceptibility to ADRs [168,169]. In addition, the increased risk of ADRs in ASD patients may occur due to several reasons. Children and adolescents with ASD are typically prescribed more than one medication to manage ASD along with comorbidities, which increases the risk of drug–drug interactions (DDIs) and the likelihood of ADRs [170]. For example, in Ritter, Hewitt and McMorris (2021) [171] study indicated that the rate of concurrent medications in this population reached up to 87%. Another reason is due to gut microbiota imbalance in ASD patients, which affects the absorption of medications and alters their pharmacokinetics [172]. Additionally, most ASD patients follow restricted diets (e.g., gluten-free), which contributes to a deficiency in co-factors (e.g., zinc, selenium) that negatively affects the metabolism of medications and alters their safety profiles [173]. Notably, ASD is linked to thyroid and adrenal dysfunction, which may potentiate endocrine-related ADR [174]. Similarly, a relationship exists between ASD and developed metabolic ADRs [33]. HCPs and caregivers also face difficulties differentiating between ADRs and ASD symptoms, such as weight gain due to physical inactivity [175,176,177]. However, there is currently no standardised framework for assessing and controlling this factor, which may contribute to variability and inconsistent ADR reporting across studies. This lack of standardisation highlights the need for an ASD-specific approach to evaluate ADR susceptibility rather than relying solely on data from the general population of mental disorders and overlooking the potential impact of ASD on ADR prevalence [178].

## 4. Research Gap and Future Directions

With the increasing global prevalence of heterogeneous diseases such as ASD and the use of AAPs as first-line treatments, the need for guidelines that consider population variation has become essential [157]. Until now, the DPWG, Clinical Pharmacogenetics Implementation Consortium (CPIC), and CAMESA have been the only organisations developing monitoring guidelines that considered individual variation within the population [49,56,154].

Over the last five years, only four studies Al-Huseini et al. (2022), Alsabhan et al. (2024), Shilbayeh and Adeen (2023), and Makary et al. (2023)—[35,179,180,181]—from the Middle Eastern regions have examined the prevalence of metabolic or endocrine ADRs associated with AAPs. Furthermore, thyroid dysfunction remains unexplored in the Middle East and is rarely investigated in other regions [50]. However, current evidence remains limited, particularly regarding population-specific ADR prevalence in paediatric patients with ASD, especially within the Middle Eastern population. This underscores the urgent need for further research and the development of tailored monitoring guidelines [157].

## 5. Clinical Implications

The study of the prevalence of metabolic and endocrine ADRs associated with AAPs in paediatric populations with ASD across different countries improves medication safety. Risk assessment helps identify high-risk populations and highlights variability in ADRs due to ethnicity, age, gender, disease, and healthcare-related factors [182,183]. As a result, this study encourages personalised treatment approaches and supports the development of tailored monitoring guidelines based on regional and individual risk profiles, including age and specific disease [184]. Additionally, it helps HCPs select safer AAPs based on the prevalence of ADRs in susceptible individuals based on their unique characteristics, thereby improving prescribing practices [185]. This study also contributes to the early detection and optimised monitoring of ADRs to prevent long-term complications, such as type 2 diabetes and cardiovascular diseases [186]. Moreover, it underscores the impact of healthcare infrastructure (e.g., spontaneous reporting systems) on the reporting and management of ADRs [163].

## 6. Conclusions

AAPs have remained the first line of treatment for paediatric patients’ irritability with ASD due to their efficacy. However, AAPs use in ASD is associated with metabolic and endocrine ADRs that contribute to public health concerns. Although data on the prevalence of these ADRs inform current monitoring guidelines, such guidelines often rely on findings from specific populations (e.g., UK, USA) without accounting for inter-individual diversity factors such as ethnicity and age.

In the future, tailored monitoring guidelines for paediatric patients with heterogeneous disorders like ASD, particularly in underrepresented populations such as those in the Middle East, should be considered in clinical practice and research. This would support developing more personalised therapies, aiming to avoid both over-monitoring of rare or non-serious ADRs and under-monitoring serious ADRs not currently included in guidelines. Taken together this would promote the safer use of AAPs in paediatric populations with ASD.

## Figures and Tables

**Table 1:** The classification of atypical antipsychotics (AAPs) ADRs based on the Hartwig scale.

Level	Severity	Definition	Example
1	Mild	ADR is detected without requiring discontinuation of medication or intervention.	Nausea, vomiting, constipation, dizziness, and headache
2	Mild	ADR detected, leading to discontinuation of medication but without need for intervention.	Sedation, extrapyramidal side effects
3	Moderate	ADR detected, requiring medication discontinuation or modification but no need for prolonged hospitalisation.	Weight gain
4	Moderate	ADR is detected and results in prolonged hospitalisation.	Hyperglycaemia
5	Severe	ADR is detected and leads to life-threatening situations or temporary disability.	Hyperlipidaemia
6	Severe	ADR contributes to permanent disability.	Hyperprolactinemia
7	Severe	ADR results in death.	Myocarditis

**Table 2:** Summary of pharmacokinetics of AAPs in paediatric populations. Abbreviation: cmax: maximum plasma concentration, F: female, ↑: increase, ↓: decrease, X: no effect, V_d_; volume distribution, PPB: plasma protein binding, t_1/2_: half-life, UGT: uridine diphosphate glucuronosyltransferase, ^a^: not effected by low-fat meal, ^b^: no active metabolites, PM: Poor metabolizer, EM: extensive metabolizers.

Drug/PK	Absorption			Distribution		Metabolism	Excretion	
	c_max_ (nM)	Bioavailability (%)	Food Effect	V_d_ (L/kg)	PPB (%)	Enzyme(s)	t_1/2_ (Hour)	Route
Risperidone	36.5	70	X	1–2	90	*CYP2D6* -With *CYP2D6*-inhibitors: ↓ by 55%	Risperidone and 9-OH-risperidone: 20 -PM: 20 -EM: 3	Urine: 70% Faeces: 14%
Aripiprazole	240.8 -F: ↑ by 11%	87	X	~5	99	*CYP2D6*	Aripiprazole: 75 Dehydroaripiprazole: 94 -PM: 150	Urine: 30% Faeces: 55%
Olanzapine	48	≥65	X	21	93	*CYP1A2* *CYP2D6* UGT1 -F: ↓ by 30% -With *CYP1A2* inducer (smoker): ↑ by 40%	30–52 ^b^	Urine: 57% Faeces:30%
Quetiapine	1291.4	10	X ^a^	6–14	83	*CYP3A4* -Hepatic impairment: dose ↓ by 0.1–0.5-fold	Quetiapine: 6 -*N*-desalkyl quetiapine: 12 -*CYP3A*4 inhibitor: ↑ by 92%	Urine: 73% Faeces: 20%
Lurasidone	60.9 -*CYP3A4* inhibitor: ↑ by 7 times	19 September	↑ by 2–3 times	2.4–20	99	*CYP3A4* -*CYP3A4* inhibitor: contraindication	lurasidone: 29 ID-14283: ↓ by 2.5 times	Urine: 9% Faeces: 80%
Ziprasidone	121 -F: ↑ by 25% -*CYP3A4* inhibitor: ↑ by 34%	60	Increased	1.5	99	glutathione and aldehyde oxidase *CYP3A4* (lesser extent) -*CYP3A4* inhibitor: monitoring therapy	5	Urine: 20% Faeces:66%

## Data Availability

Not applicable.

## Abbreviations

The following abbreviations are used in this manuscript:

AAPs	Atypical antipsychotics
ADRs	Adverse drug reactions
AgRP	Agouti-related protein
AMPK	AMP-activated protein kinase
ASD	Autism spectrum disorder
AUC	Area under the curve
BMI	Body mass index
CAMESA	Canadian Alliance for Monitoring Effectiveness and Safety of Antipsychotics
Cmax	Maximum plasma concentration
CNS	Central nervous system
CI	Confidence interval
CPIC	Clinical Pharmacogenetics Implementation Consortium
C/D	Concentration-to-dosage
D_2_	Dopamine D2
D2R	Dopamine receptor D_2_
DDIs	Drug–drug interactions
DPWG	Dutch Pharmacogenetics Working Group
DSM-IV	Diagnostic and Statistical Manual of Mental Disorders—Fourth Edition
EMA	European Medicines Agency and the Medicines
EMs	Extensive metabolizers
ERK	Extracellular signal-regulated kinase
FDA	Food and Drug Administration
FSH	Follicle-stimulating hormone
G6Pase	Glucose-6-Phosphatase
GCGR	Glucagon receptor
GFR	Glomerular filtration rate
GLUT4	Glucose transporter type 4
GnRH	Gonadotrophin-releasing hormone
H_1_, H1R	Histamine 1 Receptor
HCPs	Healthcare professionals
HPT	Hypothalamic-pituitary-thyroid
ICD-10	International Classification of Diseases, 10th edition
IL-6/2	Interleukin-6/2
IRS	Insulin receptor substrate
LH	Luteinizing hormone
M_1_	Muscarinic acetylcholine
MD	Mean difference
medianD	Median difference
MHRA	Healthcare Products Regulatory Agency
MoA	Mechanism of Action
mTOR	Mammalian target of rapamycin
NPY	Neuropeptide Y
OR	Odds ratio
P-gp	P-glycoprotein
PEPCK	Phosphoenolpyruvate carboxykinase
PI3K	Phosphoinositide 3-kinase proteins
PMs	Poor metabolizers
POMC	Pro-opiomelanocortin
PPARγ	Peroxisome proliferator-activated receptor-γ
PPB	Plasma protein binding
SFDA	Saudi Food and Drug Authority
SMD	Standardised mean difference
SREBP1/2	Sterol regulatory element-binding protein 1/2
T3	Triiodothyronine
T4	Thyroxine
t_1/2_	Half-life
TNF-α	Tumour necrosis factor-alpha
TRH	Thyrotropin-releasing hormone
TSH	Thyroid-stimulating hormone
UGT	Uridine diphosphate glucuronosyltransferase
V_d_	Volume distribution
WHO	World Health Organization
α-MSH	α-melanocyte stimulating hormone
α1/α2	alpha-1/2 adrenergic
5-HT_1_A/_2_A	5-Hydroxytryptamine (Serotonin) 1A/2A subtype
5-HT2C	5-Hydroxytryptamine (Serotonin) 2C subtype

## References

[1] Hodges H., Fealko C., Soares N. (2020). Autism spectrum disorder: Definition, epidemiology, causes, and clinical evaluation. Transl. Pediatr..

[2] O’Nions E., Petersen I., Buckman J.E., Charlton R., Cooper C., Corbett A., Happé F., Manthorpe J., Richards M., Saunders R. (2023). Autism in England: Assessing underdiagnosis in a population-based cohort study of prospectively collected primary care data. Lancet Reg. Health Eur..

[3] AlBatti T.H., Alsaghan L.B., Alsharif M.F., Alharbi J.S., BinOmair A.I., Alghurair H.A., Aleissa G.A., Bashiri F.A. (2022). Prevalence of autism spectrum disorder among Saudi children between 2 and 4 years old in Riyadh. Asian J. Psychiatry.

[4] Maenner M.J. (2023). Prevalence and characteristics of autism spectrum disorder among children aged 8 years—Autism and developmental disabilities monitoring network, 11 sites, United States, 2020. MMWR Surveill. Summ..

[5] Hassan A. (2021). Arab views on autism. Encyclopedia of Autism Spectrum Disorders.

[6] Davico C., Secci I., Vendrametto V., Vitiello B. (2023). Pharmacological treatments in autism spectrum disorder: A narrative review. J. Psychopathol..

[7] Zhou M.S., Nasir M., Farhat L.C., Kook M., Artukoglu B.B., Bloch M.H. (2021). Meta-analysis: Pharmacologic treatment of restricted and repetitive behaviors in autism spectrum disorders. J. Am. Acad. Child Adolesc. Psychiatry.

[8] de Pablo G.S., Jorda C.P., Vaquerizo-Serrano J., Moreno C., Cabras A., Arango C., Hernández P., Veenstra-VanderWeele J., Simonoff E., Fusar-Poli P. (2023). Systematic review and meta-analysis: Efficacy of pharmacological interventions for irritability and emotional dysregulation in autism spectrum disorder and predictors of response. J. Am. Acad. Child Adolesc. Psychiatry.

[9] Vita G., Nöhles V.B., Ostuzzi G., Barbui C., Tedeschi F., Heuer F.H., Keller A., DelBello M.P., Welge J.A., Blom T.J. (2025). Systematic Review and Network Meta-Analysis: Efficacy and Safety of Antipsychotics vs. Antiepileptics or Lithium for Acute Mania in Children and Adolescents. J. Am. Acad. Child Adolesc. Psychiatry.

[10] Persico A.M., Ricciardello A., Lamberti M., Turriziani L., Cucinotta F., Brogna C., Vitiello B., Arango C. (2021). The pediatric psychopharmacology of autism spectrum disorder: A systematic review-Part I: The past and the present. Prog. Neuropsychopharmacol. Biol. Psychiatry.

[11] Alsayouf H.A. (2024). Growing evidence of pharmacotherapy effectiveness in managing attention-deficit/hyperactivity disorder in young children with or without autism spectrum disorder: A minireview. Front. Psychiatry.

[12] D’Alò G.L., De Crescenzo F., Amato L., Cruciani F., Davoli M., Fulceri F., Minozzi S., Mitrova Z., Morgano G.P., Nardocci F. (2021). Impact of antipsychotics in children and adolescents with autism spectrum disorder: A systematic review and meta-analysis. Health Qual. Life Outcomes..

[13] Fonseca M., Carmo F., Martel F. (2023). Metabolic effects of atypical antipsychotics: Molecular targets. J. Neuroendocrinol..

[14] Jensen K.G. (2022). Severe hyperprolactinemia during lurasidone treatment in a 16-year old girl with schizophrenia–a case report. Scand. J. Child Adolesc. Psychiatry Psychol..

[15] Carli M., Kolachalam S., Longoni B., Pintaudi A., Baldini M., Aringhieri S., Fasciani I., Annibale P., Maggio R., Scarselli M. (2021). Atypical antipsychotics and metabolic syndrome: From molecular mechanisms to clinical differences. Pharmaceuticals.

[16] Jiang Y., Ni W. (2015). Estimating the impact of adherence to and persistence with atypical antipsychotic therapy on health care costs and risk of hospitalization. Pharmacother. J. Hum. Pharmacol. Drug Ther..

[17] Panagioti M.H.A., Planner C., Dhingra N., Gupta N. (2024). Global Burden of Preventable Medication-Related Harm in Health Care: A Systematic Review.

[18] Robinson M., Amare M. (2023). Adverse drug reactions. Anaesth. Intensive Care Med..

[19] Lee L.M., Carias D.C., Gosser R., Hannah A., Stephens S., Templeman W.A. (2022). ASHP guidelines on adverse drug reaction monitoring and reporting. Am. J. Health Syst. Pharm..

[20] Alenzi K.A., Alanazi N.S., Almalki M., Alatawi F.O. (2022). The evaluation of adverse drug reactions in Saudi Arabia: A retrospective observational study. Saudi Pharm. J..

[21] Aronson J.K. (2023). When I use a word… Medical definitions: Adverse events, effects, and reactions. Br. Med. J..

[22] World Health Organization (2002). Safety of Medicines: A Guide to Detecting and Reporting Adverse Drug Reactions: Why Health Professionals Need to Take Action.

[23] Uitvlugt E.B., Janssen M.J., Siegert C.E., Kneepkens E.L., van den Bemt B.J., van den Bemt P.M., Karapinar-Çarkit F. (2021). Medication-related hospital readmissions within 30 days of discharge: Prevalence, preventability, type of medication errors and risk factors. Front. Pharmacol..

[24] Khan M.A.A., Hamid S., Babar Z.-U.-D. (2023). Pharmacovigilance in high-income countries: Current developments and a review of literature. Pharmacy.

[25] Osanlou R., Walker L., Hughes D.A., Burnside G., Pirmohamed M. (2022). Adverse drug reactions, multimorbidity and polypharmacy: A prospective analysis of 1 month of medical admissions. BMJ Open.

[26] Abu Esba L.C., Al Mardawi G., AlJasser M.I., Aljohani B., Abu Alburak A. (2021). Adverse drug reactions spontaneously reported at a tertiary care hospital and preventable measures implemented. J. Clin. Pharm. Ther..

[27] Syahfitri H. (2021). Adverse Drug Reactions of Atypical Antipsychotics: A Review. IOSR J. Pharm..

[28] Matijević B., Delalić Đ., Meštrović D., Petrinović M., Jug J., Prkačin I. (2022). Side-effects of Medications in Emergency Medicine. Cardiol. Croat..

[29] Srisuriyachanchai W., Cox A.R., Kampichit S., Jarernsiripornkul N. (2023). Severity and management of adverse drug reactions reported by patients and healthcare professionals: A cross-sectional survey. Int. J. Environ. Res. Public Health.

[30] Palanivel M., Suresh A., Palaniappan D.R., Srinivasan D. (2024). A Step towards Patient Safety by Comparing a Trigger Tool with Pre-Existing Tools in the Pharmacovigilance. Indian. J. Pharm. Pract..

[31] Biswas M., Vanwong N., Sukasem C. (2022). Pharmacogenomics in clinical practice to prevent risperidone-induced hyperprolactinemia in autism spectrum disorder. Pharmacogenomics.

[32] Xiao T., Hu J.Q., Liu S.J., Lu H.Q., Li X.L., Kong W., Huang S.Q., Zhu X.Q., Zhang M., Lu H.Y. (2022). Population pharmacokinetics and dosing optimization of olanzapine in Chinese paediatric patients: Based on the impact of sex and concomitant valproate on clearance. J. Clin. Pharm. Ther..

[33] Chieh A.Y., Bryant B.M., Kim J.W., Li L. (2021). Systematic review investigating the relationship between autism spectrum disorder and metabolic dysfunction. Res. Autism Spectr. Disord..

[34] Goltz J., Ivanov I., Rice T.R. (2021). Second generation antipsychotic-induced weight gain in youth with autism spectrum disorders: A brief review of mechanisms, monitoring practices, and indicated treatments. Int. J. Dev. Disabil..

[35] Alsabhan J.F., Al Backer N.B., Hassan F.M., Albaker A.B., Assiry G. (2024). Metabolic Side Effects of Risperidone in Pediatric Patients with Neurological Disorders: A Prospective Cohort Study. J. Clin. Med..

[36] Carnovale C., Battini V., Santoro C., Riccio M.P., Carucci S., Nobile M., Formisano P., Bravaccio C., Zuddas A., Clementi E. (2024). Umbrella review: Association between antipsychotic drugs and metabolic syndrome hallmarks in children and adolescents. J. Am. Acad. Child Adolesc. Psychiatry.

[37] Arango C., Ng-Mak D., Finn E., Byrne A., Loebel A. (2020). Lurasidone compared to other atypical antipsychotic monotherapies for adolescent schizophrenia: A systematic literature review and network meta-analysis. Eur. Child Adolesc. Psychiatry.

[38] Pagsberg A.K., Tarp S., Glintborg D., Stenstrøm A.D., Fink-Jensen A., Correll C.U., Christensen R. (2017). Acute antipsychotic treatment of children and adolescents with schizophrenia-spectrum disorders: A systematic review and network meta-analysis. J. Am. Acad. Child Adolesc. Psychiatry.

[39] DelBello M.P., Kadakia A., Heller V., Singh R., Hagi K., Nosaka T., Loebel A. (2022). Systematic review and network meta-analysis: Efficacy and safety of second-generation antipsychotics in youths with bipolar depression. J. Am. Acad. Child Adolesc. Psychiatry.

[40] Kim M.J., Lee Y.J., Choe Y., Shin C.H., Lee Y.A. (2024). Predictors for thyroid dysfunction after discontinuation of levothyroxine in children and adolescents with Hashimoto thyroiditis. Ann. Pediatr. Endocrinol. Metab..

[41] Koch M.T., Carlson H.E., Kazimi M.M., Correll C.U. (2023). Antipsychotic-related prolactin levels and sexual dysfunction in mentally ill youth: A 3-month cohort study. J. Am. Acad. Child Adolesc. Psychiatry.

[42] Krøigaard S.M., Clemmensen L., Tarp S., Pagsberg A.K. (2022). A meta-analysis of antipsychotic-induced hypo-and hyperprolactinemia in children and adolescents. J. Child Adolesc. Psychopharmacol..

[43] Khan A.A., Sharma R., Ata F., Khalil S.K., Aldien A.S., Hasnain M., Sadiq A., Bilal A.B.I., Mirza W. (2025). Systematic review of the association between thyroid disorders and hyperprolactinemia. Thyroid. Res..

[44] Menard M.-L., Thümmler S., Giannitelli M., Cruzel C., Bonnot O., Cohen D., Askenazy F., Boublil M., Chambry J., Charvet D. (2019). Incidence of adverse events in antipsychotic-naïve children and adolescents treated with antipsychotic drugs: Results of a multicenter naturalistic study (ETAPE). Eur. Neuropsychopharmacol..

[45] Fraguas D., Merchán-Naranjo J., Laita P., Parellada M., Moreno D., Ruiz-Sancho A., Cifuentes A., Giráldez M., Arango C. (2008). Metabolic and hormonal side effects in children and adolescents treated with second-generation antipsychotics. J. Clin. Psychiatry.

[46] Zhang J.-X., Li X. (2020). Changes in serum thyroid hormone levels in psychiatric patients treated with second-generation antipsychotics. Endokrynol. Pol..

[47] Kelly D.L., Conley R.R. (2005). Thyroid function in treatment-resistant schizophrenia patients treated with quetiapine, risperidone, or fluphenazine. J. Clin. Psychiatry.

[48] Santos N.C., Costa P., Ruano D., Macedo A., Soares M.J., Valente J., Pereira A.T., Azevedo M.H., Palha J.A. (2012). Revisiting thyroid hormones in schizophrenia. J. Thyroid. Res..

[49] Jazi S., Ben-Amor L., Abadie P., Menard M.-L., Choquette R., Berthiaume C., Mottron L., Ilies D. (2021). Long-term metabolic monitoring of youths treated with second-generation antipsychotics 5 years after publication of the CAMESA guidelines are we making Progress? Surveillance Métabolique à long Terme des Jeunes Traités par Antipsychotiques de Deuxième Génération, Cinq ans Après la publication des Lignes Directrices Camesa: Faisons-nous des Progrès?. Can. J. Psychiatry.

[50] Khoodoruth M.A.S., Abdo A.K.A., Ouanes S. (2022). Quetiapine-induced thyroid dysfunction: A systematic review. J. Clin. Pharmacol..

[51] Alvarez-Herrera S., Escamilla R., Medina-Contreras O., Saracco R., Flores Y., Hurtado-Alvarado G., Maldonado-García J.L., Becerril-Villanueva E., Pérez-Sánchez G., Pavón L. (2020). Immunoendocrine peripheral effects induced by atypical antipsychotics. Front. Endocrinol..

[52] Findling R.L., Pathak S., Earley W.R., Liu S., DelBello M.P. (2014). Efficacy and safety of extended-release quetiapine fumarate in youth with bipolar depression: An 8 week, double-blind, placebo-controlled trial. J. Child Adolesc. Psychopharmacol..

[53] Beaulieu J.-M., Gainetdinov R.R. (2011). The physiology, signaling, and pharmacology of dopamine receptors. Pharmacol. Rev..

[54] Chien H.-Y., Chen S.-M., Li W.-C. (2023). Dopamine receptor agonists mechanism of actions on glucose lowering and their connections with prolactin actions. Front. Clin. Diabetes Healthc..

[55] Libowitz M.R., Nurmi E.L. (2021). The burden of antipsychotic-induced weight gain and metabolic syndrome in children. Front. Psychiatry.

[56] Soria-Chacartegui P., Villapalos-García G., Zubiaur P., Abad-Santos F., Koller D. (2021). Genetic polymorphisms associated with the pharmacokinetics, pharmacodynamics and adverse effects of olanzapine, aripiprazole and risperidone. Front. Pharmacol..

[57] Al-Zoairy R., Pedrini M.T., Khan M.I., Engl J., Tschoner A., Ebenbichler C., Gstraunthaler G., Salzmann K., Bakry R., Niederwanger A. (2017). Serotonin improves glucose metabolism by Serotonylation of the small GTPase Rab4 in L6 skeletal muscle cells. Diabetol. Metab. Syndr..

[58] Ferreira V., Grajales D., Valverde Á.M. (2020). Adipose tissue as a target for second-generation (atypical) antipsychotics: A molecular view. Biochim. Biophys. Acta Mol. Cell Biol. Lipids.

[59] Wang K.-Y., Tanimoto A., Yamada S., Guo X., Ding Y., Watanabe T., Watanabe T., Kohno K., Hirano K.-I., Tsukada H. (2010). Histamine regulation in glucose and lipid metabolism via histamine receptors: Model for nonalcoholic steatohepatitis in mice. Am. J. Pathol..

[60] Ren L., Zhou X., Huang X., Wang C., Li Y. (2019). The IRS/PI3K/Akt signaling pathway mediates olanzapine-induced hepatic insulin resistance in male rats. Life Sci..

[61] Nagata M., Yokooji T., Nakai T., Miura Y., Tomita T., Taogoshi T., Sugimoto Y., Matsuo H. (2019). Blockade of multiple monoamines receptors reduce insulin secretion from pancreatic β-cells. Sci. Rep..

[62] Zhao S., Lin Q., Xiong W., Li L., Straub L., Zhang D., Zapata R., Zhu Q., Sun X.-N., Zhang Z. (2023). Hyperleptinemia contributes to antipsychotic drug–associated obesity and metabolic disorders. Sci. Transl. Med..

[63] Khan M.M., Khan Z.A., Khan M.A. (2024). Metabolic complications of psychotropic medications in psychiatric disorders: Emerging role of de novo lipogenesis and therapeutic consideration. World J. Psychiatry.

[64] Anjum F., Ali M.M., Jakoby M., Williams V. (2023). Abstract# 1401109: Quetiapine-induced Hypoglycemia in an Elderly Non-diabetic Patient with Dementia. Endocr. Pract..

[65] Vasiliu O. (2023). Therapeutic management of atypical antipsychotic-related metabolic dysfunctions using GLP-1 receptor agonists: A systematic review. Exp. Ther. Med..

[66] Ebrahimian Z., Razavi B.M., Mousavi Shaegh S.A., Hosseinzadeh H. (2025). Exploring the therapeutic potential of chlorogenic acid in alleviating olanzapine-induced metabolic syndrome in rats: A key role of hypothalamic satiety proteins. Nutr. Neurosci..

[67] Ballon J.S., Pajvani U., Freyberg Z., Leibel R.L., Lieberman J.A. (2014). Molecular pathophysiology of metabolic effects of antipsychotic medications. Trends Endocrinol. Metab..

[68] Zhuo C., Xu Y., Hou W., Chen J., Li Q., Liu Z., Dou G., Sun Y., Li R., Ma X. (2022). Mechanistic/mammalian target of rapamycin and side effects of antipsychotics: Insights into mechanisms and implications for therapy. Transl. Psychiatry.

[69] Sobiś J., Kunert Ł., Rykaczewska-Czerwińska M., Świętochowska E., Gorczyca P. (2022). The effect of aripiprazole on leptin levels of patients with chronic schizophrenia and a comparison of leptin, acute phase protein, and cytokine levels with regard to body mass and body composition indexes. Endokrynol. Pol..

[70] Zhao X., Zhu W., Bu Y., Li J., Hao Y., Bi Y. (2024). Effects of 6-week olanzapine treatment on serum IL-2, IL-4, IL-8, IL-10, and TNF-α levels in drug-naive individuals with first-episode schizophrenia. BMC Psychiatry.

[71] Pillinger T., McCutcheon R.A., Vano L., Mizuno Y., Arumuham A., Hindley G., Beck K., Natesan S., Efthimiou O., Cipriani A. (2020). Comparative effects of 18 antipsychotics on metabolic function in patients with schizophrenia, predictors of metabolic dysregulation, and association with psychopathology: A systematic review and network meta-analysis. Lancet Psychiatry.

[72] Spina E., Barbieri M.A., Cicala G., de Leon J. (2020). Clinically relevant interactions between atypical antipsychotics and anti-infective agents. Pharmaceuticals.

[73] Edinoff A.N., Silverblatt N.S., Vervaeke H.E., Horton C.C., Girma E., Kaye A.D., Kaye A., Kaye J.S., Garcia A.J., Neuchat E.E. (2021). Hyperprolactinemia, clinical considerations, and infertility in women on antipsychotic medications. Psychopharmacol. Bull..

[74] Jumaili W.A., Muzwagi A. (2022). Review of the Long-Term Effect of the Atypical Antipsychotic Medication on the Bone Mineral Density of the Pediatric Patient with Consideration of Autism Spectrum Disorder. J. Pharmacol. Pharmacother..

[75] Stojkovic M., Radmanovic B., Jovanovic M., Janjic V., Muric N., Ristic D.I. (2022). Risperidone induced hyperprolactinemia: From basic to clinical studies. Front. Psychiatry.

[76] Baykara H.B., Güney S.A., Avcil S., Buran B.Ş., Cıray R.O., Ermis C., Inal N. (2024). Safety of Atypical Antipsychotics in a Child and Adolescent Inpatient Setting: A Naturalistic Study. Psychiatry Clin. Psychopharmacol..

[77] Mandal S., Chowdhury S., Saha P.K., Chowdhury J. (2024). Hyperprolactinemia vs. Thyroid Autoimmunity: Which is a Greater Concern with Atypical Antipsychotics?. Res. Squar.

[78] Pei X., Du X., Lu H., Liu D. (2024). Successful Rescue of Hypotension Shock Induced by Quetiapine: A Case Report. Ann. Clin. Case Rep..

[79] Theilade S., Christensen M.B., Vilsbøll T., Knop F.K. (2021). An overview of obesity mechanisms in humans: Endocrine regulation of food intake, eating behaviour and common determinants of body weight. Diabetes Obes. Metab..

[80] Kong L., Shen Y., Hu S., Lai J. (2024). The impact of quetiapine monotherapy or in combination with lithium on the thyroid function in patients with bipolar depression: A retrospective study. CNS Neurosci. Ther..

[81] Liang J., Ringeling L.T., Hermans R.A., Bayraktar I., Bosch T.M., Egberts K.M., Kloosterboer S.M., de Winter B., Dierckx B., Koch B.C. (2023). Clinical pharmacokinetics of antipsychotics in pediatric populations: A scoping review focusing on dosing regimen. Expert. Opin. Drug Metab. Toxicol..

[82] Chen F., Liu H., Wang B., Yang L., Cai W., Jiao Z., Yang Z., Chen Y., Quan Y., Xiang X. (2020). Physiologically based pharmacokinetic modeling to understand the absorption of risperidone orodispersible film. Front. Pharmacol..

[83] Townsend R.W. (2021). Low Dose Risperidone Every 3.8 Hours: Superior Efficacy In Treatment of Bipolar Disorders. Res. Sq..

[84] Liu Y., Zhang M.-q., Jia J.-y., Liu Y.-m., Liu G.-y., Li S.-j., Wang W., Weng L.-p., Yu C. (2013). Bioequivalence and pharmacokinetic evaluation of two formulations of risperidone 2 mg: An open-label, single-dose, fasting, randomized-sequence, two-way crossover study in healthy male Chinese volunteers. Drugs R D.

[85] de Leon J. (2020). Personalizing dosing of risperidone, paliperidone and clozapine using therapeutic drug monitoring and pharmacogenetics. Neuropharmacology.

[86] Caccia S. (2013). Safety and pharmacokinetics of atypical antipsychotics in children and adolescents. Pediatr. Drugs..

[87] Stahl S.M., Strawn J.R. (2024). Prescriber’s Guide-Children and Adolescents: Stahl’s Essential Psychopharmacology.

[88] de Leon J. (2022). Precision psychiatry: The complexity of personalizing antipsychotic dosing. Eur. Neuropsychopharmacol..

[89] Lu J., Yang Y., Lu J., Wang Z., He Y., Yan Y., Fu K., Jiang W., Xu Y., Wu R. (2021). Effect of CYP2D6 polymorphisms on plasma concentration and therapeutic effect of risperidone. BMC Psychiatry.

[90] Xiang J., Xu N., Wang X., Li S., Yu Q., Liang M., Nan F., Shu S., Yan R., Zhu Y. (2021). Bioequivalence of 2 aripiprazole orally disintegrating tablets in healthy Chinese volunteers under fasting and fed conditions. Clin. Pharmacol. Drug Dev..

[91] Kumar A., Singh H., Mishra A., Mishra A.K. (2020). Aripiprazole: An FDA approved bioactive compound to treat schizophrenia-A mini review. Curr. Drug Discov. Technol..

[92] Kirino E. (2014). Profile of aripiprazole in the treatment of bipolar disorder in children and adolescents. Adolesc. Health Med. Ther..

[93] Biswas M., Vanwong N., Sukasem C. (2024). Pharmacogenomics and non-genetic factors affecting drug response in autism spectrum disorder in Thai and other populations: Current evidence and future implications. Front. Pharmacol..

[94] Egberts K., Reuter-Dang S.-Y., Fekete S., Kulpok C., Mehler-Wex C., Wewetzer C., Karwautz A., Mitterer M., Holtkamp K., Boege I. (2020). Therapeutic drug monitoring of children and adolescents treated with aripiprazole: Observational results from routine patient care. J. Neural Transm..

[95] Findling R.L., Kauffman R.E., Sallee F.R., Carson W.H., Nyilas M., Mallikaarjun S., Shoaf S.E., Forbes R.A., Boulton D.W., Pikalov A. (2008). Tolerability and pharmacokinetics of aripiprazole in children and adolescents with psychiatric disorders: An open-label, dose-escalation study. J. Clin. Psychopharmacol..

[96] Jukić M.M., Smith R.L., Molden E., Ingelman-Sundberg M. (2021). Evaluation of the CYP2D6 haplotype activity scores based on metabolic ratios of 4,700 patients treated with three different CYP2D6 substrates. Clin. Pharmacol. Ther..

[97] Shan Y., Cheung L., Zhou Y., Huang Y., Huang R.S. (2023). A systematic review on sex differences in adverse drug reactions related to psychotropic, cardiovascular, and analgesic medications. Front. Pharmacol..

[98] Xin Y., Gao L., Tuo Y., Nie G., Mei Y., Chen C., Wang J., Li S., Sun D., Qian Q. (2022). Understanding inter-individual variability in pharmacokinetics/pharmacodynamics of aripiprazole in children with tic disorders: Individualized administration based on physiological development and CYP2D6 genotypes. Front. Pharmacol..

[99] Rudå D., Jensen K.G., Decara M.S., Klauber D.G., Fagerlund B., Møllegaard J.R., Linnet K., Werge T., Correll C.U., Fink-Jensen A. (2021). CYP2D6 genotyping and antipsychotic-associated extrapyramidal adverse effects in a randomized trial of aripiprazole versus quetiapine extended release in children and adolescents, aged 12–17 years, with first episode psychosis. J. Clin. Psychopharmacol..

[100] Zubiaur P., Soria-Chacartegui P., Koller D., Navares-Gómez M., Ochoa D., Almenara S., Saiz-Rodríguez M., Mejía-Abril G., Villapalos-García G., Román M. (2021). Impact of polymorphisms in transporter and metabolizing enzyme genes on olanzapine pharmacokinetics and safety in healthy volunteers. Biomed. Pharmacother..

[101] Maharaj A.R., Wu H., Zimmerman K.O., Autmizguine J., Kalra R., Al-Uzri A., Sherwin C.M., Goldstein S.L., Watt K., Erinjeri J. (2021). Population pharmacokinetics of olanzapine in children. Br. J. Clin. Pharmacol..

[102] Penzak S.R., Hon Y.Y., Lawhorn W.D., Shirley K.L., Spratlin V., Jann M.W. (2002). Influence of ritonavir on olanzapine pharmacokinetics in healthy volunteers. J. Clin. Psychopharmacol..

[103] Kolli P., Kelley G., Rosales M., Faden J., Serdenes R. (2023). Olanzapine pharmacokinetics: A clinical review of current insights and remaining questions. Pharmgenomics Pers. Med..

[104] Erickson-Ridout K.K., Zhu J., Lazarus P. (2011). Olanzapine metabolism and the significance of UGT1A448V and UGT2B1067Y variants. Pharmacogenet Genom..

[105] Mao J.-H., Han L., Liu X.-Q., Jiao Z. (2023). Significant predictors for olanzapine pharmacokinetics: A systematic review of population pharmacokinetic studies. Expert. Rev. Clin. Pharmacol..

[106] Fekete S., Wewetzer C., Mehler-Wex C., Holtkamp K., Burger R., Reichert S., Taurines R., Romanos M., Gerlach M., Egberts K. (2017). Therapeutic drug monitoring in children and adolescents under pharmacotherapy with olanzapine in daily clinical practice. Ther. Drug Monit..

[107] Grothe D.R., Calis K.A., Jacobsen L., Kumra S., DeVane C.L., Rapoport J.L., Bergstrom R.F., Kurtz D.L. (2000). Olanzapine pharmacokinetics in pediatric and adolescent inpatients with childhood-onset schizophrenia. J. Clin. Psychopharmacol..

[108] Aichhorn W., Marksteiner J., Walch T., Zernig G., Hinterhuber H., Stuppaeck C., Kemmler G. (2007). Age and gender effects on olanzapine and risperidone plasma concentrations in children and adolescents. J. Child Adolesc. Psychopharmacol..

[109] Keepers G.A., Fochtmann L.J., Anzia J.M., Benjamin S., Lyness J.M., Mojtabai R., Servis M., Walaszek A., Buckley P., Lenzenweger M.F. (2020). The American Psychiatric Association practice guideline for the treatment of patients with schizophrenia. Am. J. Psychiatry.

[110] Blacker C.J. (2020). Clinical issues to consider for clozapine patients who vape: A case illustration. Focus.

[111] Yükselmiş U., Akçay M., Alomari O., Yılmaz M.K. (2024). A case report of combined hemoperfusion and hemodiafiltration utilization in pediatric severe Quetiapine poisoning. J. Med. Surg. Public Health.

[112] Ortega-Ruiz M., Soria-Chacartegui P., Villapalos-García G., Abad-Santos F., Zubiaur P. (2022). The pharmacogenetics of treatment with quetiapine. Future Pharmacol..

[113] Joshi K., Rao S., Mehta S. (2025). A Review of Pharmacokinetic and Pharmacodynamic Properties of Quetiapine IR and XR: Insights and Clinical Practice Implications. Cureus.

[114] Bertol E., Vaiano F., Argo A., Zerbo S., Trignano C., Protani S., Favretto D. (2021). Overdose of quetiapine—A case report with QT prolongation. Toxics.

[115] Shnayder N.A., Abdyrakhmanova A.K., Nasyrova R.F. (2022). Oxidation of antipsychotics. Encyclopedia.

[116] Castberg I., Skogvoll E., Spigset O. (2007). Quetiapine and drug interactions: Evidence from a routine therapeutic drug monitoring service. J. Clin. Psychiatry.

[117] Fekete S., Hiemke C., Gerlach M. (2020). Dose-related concentrations of neuroactive/psychoactive drugs expected in blood of children and adolescents. Ther. Drug Monit..

[118] McConville B.J., Arvanitis L.A., Thyrum P.T., Yeh C., Wilkinson L.A., Chaney R.O., Foster K.D., Sorter M.T., Friedman L.M., Brown K.L. (2000). Pharmacokinetics, tolerability, and clinical effectiveness of quetiapine fumarate: An open-label trial in adolescents with psychotic disorders. J. Clin. Psychiatry.

[119] Zubiaur P., Fernández-Campos P., Navares-Gómez M., Soria-Chacartegui P., Villapalos-García G., Román M., Mejía-Abril G., Ochoa D., Abad-Santos F. (2021). Variants in COMT, CYP3A5, CYP2B6, and ABCG2 alter quetiapine pharmacokinetics. Pharmaceutics.

[120] Yau K., McArthur E., Jeyakumar N., Tsobo Muanda F., Kim R.B., Clemens K.K., Wald R., Garg A.X. (2023). Adverse events with quetiapine and clarithromycin coprescription: A population-based retrospective cohort study. Health Sci. Rep..

[121] Jang J.-H., Jeong S.-H. (2025). Pharmacokinetic Prediction of Immediate-and Extended-Release Tablets for Patients with Liver Disease Using Whole Body Physiologically-Based Pharmacokinetic Modeling for the Antipsychotic Drug Quetiapine. AAPS PharmSciTech.

[122] Li K.-Y., Li X., Cheng Z.-N., Zhang B.-K., Peng W.-X., Li H.-D. (2005). Effect of erythromycin on metabolism of quetiapine in Chinese suffering from schizophrenia. Eur. J. Clin. Pharmacol..

[123] Lee S.-M., Jang J.-H., Jeong S.-H. (2024). Exploring gender differences in pharmacokinetics of central nervous system related medicines based on a systematic review approach. Naunyn-Schmiedeberg’s Arch. Pharmacol..

[124] McGrane I.R., Salyers L.A., Molinaro J.R., Munjal R.C. (2021). Roux-en-Y gastric bypass and antipsychotic therapeutic drug monitoring: Two cases. J. Pharm. Pract..

[125] Amerio A., Giacomini C., Fusar-Poli L., Aguglia A., Costanza A., Serafini G., Aguglia E., Amore M. (2021). Efficacy and safety of lurasidone in children and adolescents: Recommendations for clinical management and future research. Curr. Pharm. Des..

[126] Findling R.L., Goldman R., Chiu Y.-Y., Silva R., Jin F., Pikalov A., Loebel A. (2015). Pharmacokinetics and tolerability of lurasidone in children and adolescents with psychiatric disorders. Clin. Ther..

[127] Siwek M., Krupa A., Wasik A. (2020). Lurasidone–pharmacodynamic and pharmacokinetic properties, clinical potential and interaction risk. Pharmacother. Psychiatry Neurol..

[128] Mostafa Y.E., Metwally M.E.S., Elsebaei F. (2024). Prominently selective fluorescence approach with distinctive biopharmaceutical utility for analysis of lurasidone in human plasma and urine: Application to in vitro dissolution and content uniformity testing. Luminescence.

[129] Lin S.-K. (2022). Racial/ethnic differences in the pharmacokinetics of antipsychotics: Focusing on East Asians. J. Pers. Med..

[130] Shirley M. (2021). Lurasidone in schizophrenia in adolescents: A profile of its use. Drugs Ther. Perspect..

[131] Mole T.B., Furlong Y., Clarke R.J., Rao P., Moore J.K., Pace G., Van Odyck H., Chen W. (2022). Lurasidone for adolescents with complex mental disorders: A case series. J. Pharm. Pract..

[132] Yang Y., Wang Z., Xiao T., Ni X., Song E., Dai L., Chen Y., Lu H., Shang D., Wen Y. (2022). Preliminary Determination of the Therapeutic Reference Range of Lurasidone in Chinese Patients and Analysis of the Factors Influencing Lurasidone Dose-Corrected Concentrations. Ther. Drug Monit..

[133] Chiu Y.-Y., Ereshefsky L., Preskorn S.H., Poola N., Loebel A. (2014). Lurasidone drug-drug interaction studies: A comprehensive review. Drug Metab. Drug Interact..

[134] Paun J.S., Tank H.M., Savalia V.B., Chaitanya J.K. (2025). Ziprasidone Hydrochloride Nanosuspension for Bioavailability Enhancement: Design, Development and Evaluation. E3S Web Conf..

[135] Khan A.A., Strawn J.R., Croarkin P.E. (2010). Emerging treatment options in bipolar disorder in adolescents: Focus on ziprasidone. Adolesc. Health Med. Ther..

[136] Bao S., Yang S., Hua Z., Li J., Zang Y., Li X. (2024). Ziprasidone population pharmacokinetics and co-medication effects in Chinese patients. Naunyn-Schmiedeberg’s Arch. Pharmacol..

[137] Simon N., Torrents R., Azorin J.-M. (2022). Comorbidities and the right dose: Antipsychotics. Expert. Opin. Drug Metab. Toxicol..

[138] Sallee F.R., Miceli J.J., Tensfeldt T., Robarge L., Wilner K., Patel N.C. (2006). Single-dose pharmacokinetics and safety of ziprasidone in children and adolescents. J. Am. Acad. Child Adolesc. Psychiatry.

[139] Miceli J., Smith M., Robarge L., Morse T., Laurent A. (2000). The effects of ketoconazole on ziprasidone pharmacokinetics—A placebo-controlled crossover study in healthy volunteers. Br. J. Clin. Pharmacol..

[140] Cicala G., Barbieri M.A., Santoro V., Tata C., Colucci P.V., Vanadia F., Drago F., Russo C., Cutroneo P.M., Gagliano A. (2020). Safety and tolerability of antipsychotic drugs in pediatric patients: Data from a 1-year naturalistic study. Front. Psychiatry.

[141] Schoretsanitis G., de Leon J., Correll C.U. (2024). How can we better address the pharmacokinetics of antipsychotics in children and adolescents?. Expert. Opin. Drug Metab. Toxicol..

[142] Chapron B.D., Chapron A., Leeder J.S. (2022). Recent advances in the ontogeny of drug disposition. Br. J. Clin. Pharmacol..

[143] O’hara K. (2016). Paediatric pharmacokinetics and drug doses. Aust. Prescr..

[144] Sanyal S., Calarge C.A., Rowan P.J., Aparasu R.R., Abughosh S., Chen H. (2023). Impact of the AACAP practice parameters on the metabolic adverse event monitoring for second-generation antipsychotics (SGAs) in children and adolescents. J. Psychiatr. Res..

[145] Hoekstra S., Bartz-Johannessen C., Sinkeviciute I., Reitan S.K., Kroken R.A., Løberg E.-M., Larsen T.K., Rettenbacher M., Johnsen E., Sommer I.E. (2021). Sex differences in antipsychotic efficacy and side effects in schizophrenia spectrum disorder: Results from the BeSt InTro study. npj Schizophr..

[146] Johansen I.T., Steen N.E., Rødevand L., Werner M.C., Lunding S.H., Hjell G., Ormerod M.B., Agartz I., Melle I., Lagerberg T.V. (2022). Sex-specific associations between metabolic hormones, severe mental disorders and antipsychotic treatment. Psychoneuroendocrinology.

[147] Zucker I., Prendergast B.J. (2020). Sex differences in pharmacokinetics predict adverse drug reactions in women. Biol. Sex. Differ..

[148] Klein S.L., Morgan R. (2020). The impact of sex and gender on immunotherapy outcomes. Biol. Sex. Differ..

[149] Jovanović M., Vučićević K., Miljković B. (2020). Understanding variability in the pharmacokinetics of atypical antipsychotics–focus on clozapine, olanzapine and aripiprazole population models. Drug Metab. Rev..

[150] Brand B.A., Haveman Y.R., De Beer F., De Boer J.N., Dazzan P., Sommer I.E. (2022). Antipsychotic medication for women with schizophrenia spectrum disorders. Psychol. Med..

[151] Elmeliegy M., Vourvahis M., Guo C., Wang D.D. (2020). Effect of P-glycoprotein (P-gp) inducers on exposure of P-gp substrates: Review of clinical drug–drug interaction studies. Clin. Pharmacokinet..

[152] Watson S., Caster O., Rochon P.A., Den Ruijter H. (2019). Reported adverse drug reactions in women and men: Aggregated evidence from globally collected individual case reports during half a century. EClinicalMedicine.

[153] Shilbayeh S.A.R., Adeen I.S., Alhazmi A.S., Aldilaijan K.E., Aloyouni S.Y. (2023). Risperidone pharmacogenetics: The impact of star alleles’ predicted phenotypes on global safety in autistic children. Int. J. Pharmacol..

[154] Beunk L., Nijenhuis M., Soree B., de Boer-Veger N.J., Buunk A.-M., Guchelaar H.J., Houwink E.J., Risselada A., Rongen G.A., van Schaik R.H. (2024). Dutch Pharmacogenetics Working Group (DPWG) guideline for the gene-drug interaction between CYP2D6, CYP3A4 and CYP1A2 and antipsychotics. Eur. J. Hum. Genet..

[155] Tveito M., Molden E., Høiseth G., Correll C.U., Smith R.L. (2020). Impact of age and CYP2D6 genetics on exposure of aripiprazole and dehydroaripiprazole in patients using long-acting injectable versus oral formulation: Relevance of poor and intermediate metabolizer status. Eur. J. Clin. Pharmacol..

[156] Jukic M.M., Smith R.L., Haslemo T., Molden E., Ingelman-Sundberg M. (2019). Effect of CYP2D6 genotype on exposure and efficacy of risperidone and aripiprazole: A retrospective, cohort study. Lancet Psychiatry.

[157] Ahmed Z., Hao S., Williamson T., McMorris C.A., Bousman C.A. (2022). Psychotropic prescribing rates and pharmacogenomic testing implications for autism in the Canadian primary care sentinel surveillance network. Pharmacogenet Genom..

[158] Cui Y., Yan H., Su Y., Wang L., Lu T., Zhang D., Yue W. (2020). CYP2D6 genotype-based dose recommendations for risperidone in Asian people. Front. Pharmacol..

[159] Chamnanphon M., Vanwong N., Prommas S., Koomdee N., Sukprasong R., Rachanakul J., Nuntharadthanaphong N., Hongkaew Y., John S., Ngamsamut N. (2022). Risperidone plasma concentrations are associated with hyperprolactinemia in autism spectrum disorder children: The impact of CYP2D6 polymorphisms. Res. Autism Spectr. Disord..

[160] Merino D., Fernandez A., Gerard A.O., Ben Othman N., Rocher F., Askenazy F., Verstuyft C., Drici M.-D., Thümmler S. (2022). Adverse Drug Reactions of Olanzapine, Clozapine and Loxapine in Children and Youth: A Systematic Pharmacogenetic Review. Pharmaceuticals.

[161] Baldacci A., Saguin E., Balcerac A., Mouchabac S., Ferreri F., Gaillard R., Colas M.-D., Delacour H., Bourla A. (2023). Pharmacogenetic guidelines for psychotropic drugs: Optimizing prescriptions in clinical practice. Pharmaceutics.

[162] Dubale A.T., Tareke A.A., Butta F.W., Shibabaw A.A., Eniyew E.B., Ahmed M.H., Kassie S.Y., Demsash A.W., Chereka A.A., Dube G.N. (2024). Healthcare professionals’ willingness to utilize a mobile health application for adverse drug reaction reporting in a limited resource setting: An input for digital health, 2023. Eur. J. Obstet. Gynecol. Reprod. Biol. X.

[163] Worakunphanich W., Youngkong S., Suwankesawong W., Anderson C., Thavorncharoensap M. (2022). Comparison of patient adverse drug reaction reporting systems in nine selected countries. Int. J. Environ. Res. Public Health.

[164] Alshammari T.M. (2025). Pharmacovigilance and outcomes: Experience from Saudi Arabia narrative review. Expert. Rev. Pharmacoecon. Outcomes Res..

[165] Costa C., Abeijon P., Rodrigues D.A., Figueiras A., Herdeiro M.T., Torre C. (2023). Factors associated with underreporting of adverse drug reactions by patients: A systematic review. Int. J. Clin. Pharm..

[166] Gauci R. (2024). Digitalisation of Adverse Drug Reactions Information Source.

[167] Al-Worafi Y.M. (2023). Adverse Drug Reactions (ADRs) in Developing Countries. Handbook of Medical and Health Sciences in Developing Countries: Education, Practice, and Research.

[168] Beversdorf D.Q., Anagnostou E., Hardan A., Wang P., Erickson C.A., Frazier T.W., Veenstra-VanderWeele J. (2023). Precision medicine approaches for heterogeneous conditions such as autism spectrum disorders (The need for a biomarker exploration phase in clinical trials-Phase 2m). Front. Psychiatry.

[169] Lacivita E., Niso M., Mastromarino M., Garcia Silva A., Resch C., Zeug A., Loza M.I., Castro M., Ponimaskin E., Leopoldo M. (2021). Knowledge-based design of long-chain arylpiperazine derivatives targeting multiple serotonin receptors as potential candidates for treatment of autism spectrum disorder. ACS Chem. Neurosci..

[170] Clavenna A., Cartabia M., Fortino I., Bonati M. (2024). Drug prescription profile in children with autism spectrum disorders. Eur. J. Clin. Pharmacol..

[171] Ritter C., Hewitt K., McMorris C.A. (2021). Psychotropic polypharmacy among children and youth with autism: A systematic review. J. Child Adolesc. Psychopharmacol..

[172] Dargenio V.N., Dargenio C., Castellaneta S., De Giacomo A., Laguardia M., Schettini F., Francavilla R., Cristofori F. (2023). Intestinal barrier dysfunction and microbiota–gut–brain axis: Possible implications in the pathogenesis and treatment of autism spectrum disorder. Nutrients.

[173] Długosz A., Wróblewski M., Błaszak B., Szulc J. (2025). The Role of Nutrition, Oxidative Stress, and Trace Elements in the Pathophysiology of Autism Spectrum Disorders. Int. J. Mol. Sci..

[174] De Luca F. (2020). Endocrinological abnormalities in autism. Semin. Pediatr. Neurol..

[175] Blake K.V., Zaccaria C., Domergue F., La Mache E., Saint-Raymond A., Hidalgo-Simon A. (2014). Comparison between paediatric and adult suspected adverse drug reactions reported to the European medicines agency: Implications for pharmacovigilance. Pediatr. Drugs..

[176] Dubrall D., Leitzen S., Toni I., Stingl J., Schulz M., Schmid M., Neubert A., Sachs B. (2021). Descriptive analysis of adverse drug reaction reports in children and adolescents from Germany: Frequently reported reactions and suspected drugs. BMC Pharmacol. Toxicol..

[177] Braykova R., Toneva A. (2025). Aspects of Insulin Resistance in Children with Autism. J. IMAB..

[178] Aljead M., Qashta A., Jalal Z., Jones A.M. (2025). Review of Autism Spectrum Disorder (ASD): Epidemiology, Aetiology, Pathology, and Pharmacological Treatment. Pharmaceuticals.

[179] Al-Huseini S., Al-Barhoumi A., Al-Balushi M., Al-Hosni A., Al-Mahrouqi T., Al-Mahrizi B., Jaju S., Mirza H. (2022). Effectiveness and adverse effects of risperidone in children with autism spectrum disorder in a naturalistic clinical setting at a university hospital in Oman. Autism Res. Treat..

[180] Rahim Shilbayeh S.A., Adeen I.S. (2024). Management of autism spectrum disorder: A pilot study in saudi paediatrics. Mil. Med. Sci. Lett..

[181] Makary S., Abd El Moez K., Elsayed M., Hassan H. (2023). Second-generation antipsychotic medications and metabolic disturbance in children and adolescents. Egypt. J. Neurol. Psychiatry Neurosurg..

[182] Man K.K., Shao S.-C., Chang Y.-C., Chi M.-H., Jeong H.E., Lin S.-J., Su C.-C., Shin J.-Y., Wong K.H., Wong I.C. (2021). Cardiovascular and metabolic risk of antipsychotics in children and young adults: A multinational self-controlled case series study. Epidemiol. Psychiatr. Sci..

[183] Iasevoli F., Barone A., Buonaguro E.F., Vellucci L., de Bartolomeis A. (2020). Safety and tolerability of antipsychotic agents in neurodevelopmental disorders: A systematic review. Expert. Opin. Drug Saf..

[184] Hernandez M., Cullell N., Cendros M., Serra-Llovich A., Arranz M.J. (2024). Clinical utility and implementation of pharmacogenomics for the personalisation of antipsychotic treatments. Pharmaceutics.

[185] Mead L., Ayres A., Blake J.A., Scott J.G. (2021). Monitoring of metabolic side-effects in children and adolescents prescribed antipsychotic medication: A systematic review. Aust. N. Z. J. Psychiatry.

[186] Fekete S., Güntzel T., Egberts K., Geissler J., Neubert A., Gerlach M., Romanos M., Taurines R. (2023). Serious adverse drug reactions to antipsychotics in minors with multiple disabilities: Preventability and potential cost savings by therapeutic drug monitoring. Pharmacopsychiatry.

